# Update in Viral Infections in the Intensive Care Unit

**DOI:** 10.3389/fmed.2021.575580

**Published:** 2021-02-23

**Authors:** Paraskevi C. Fragkou, Charalampos D. Moschopoulos, Emmanouil Karofylakis, Theodoros Kelesidis, Sotirios Tsiodras

**Affiliations:** ^1^4th Department of Internal Medicine, Medical School, National and Kapodistrian University of Athens, “Attikon” University Hospital, Athens, Greece; ^2^Department of Medicine, David Geffen School of Medicine, University of California, Los Angeles, Los Angeles, CA, United States

**Keywords:** viral infections, reactivation, respiratory tract infection, intensive care unit, critically ill, critical care, neurologic syndrome, shock

## Abstract

The advent of highly sensitive molecular diagnostic techniques has improved our ability to detect viral pathogens leading to severe and often fatal infections that require admission to the Intensive Care Unit (ICU). Viral infections in the ICU have pleomorphic clinical presentations including pneumonia, acute respiratory distress syndrome, respiratory failure, central or peripheral nervous system manifestations, and viral-induced shock. Besides *de novo* infections, certain viruses fall into latency and can be reactivated in both immunosuppressed and immunocompetent critically ill patients. Depending on the viral strain, transmission occurs either directly through contact with infectious materials and large droplets, or indirectly through suspended air particles (airborne transmission of droplet nuclei). Many viruses can efficiently spread within hospital environment leading to in-hospital outbreaks, sometimes with high rates of mortality and morbidity, thus infection control measures are of paramount importance. Despite the advances in detecting viral pathogens, limited progress has been made in antiviral treatments, contributing to unexpectedly high rates of unfavorable outcomes. Herein, we review the most updated data on epidemiology, common clinical features, diagnosis, pathogenesis, treatment and prevention of severe community- and hospital-acquired viral infections in the ICU settings.

## Introduction

The incidence of viral infections in critical care settings varies widely across different studies and geographical regions, but it has unambiguously increased over the last years as modern diagnostics have become more sensitive, rapid and accurate (1, 2). The advent of sophisticated molecular tools has facilitated the detection of emerging viral pathogens, such as the novel Severe Acute Respiratory Syndrome coronavirus 2 (SARS-CoV-2), leading to the identification of viruses as culprits in previously undiagnosed conditions. Due to the fact that antiviral therapies are still scarce, the mainstay of treatment for many life-threatening viral infections remains supportive, and therefore critical care is of paramount importance for the management of severe viral infections (3).

In this narrative review we aim to present an updated overview of the epidemiology, geographic distribution, clinical features, diagnosis, treatment and specific preventative measures for viral pathogens associated with (i) severe community acquired respiratory tract infections and nosocomial pneumonia (ii) neurological and neuromuscular diseases requiring intensive care unit (ICU) admission, (iii) different types of shock and multi-organ injury, and finally, (iv) re-activation, in critically ill patients.

## Epidemiology of Viral Pathogens Causing Critical Illness

### Respiratory Viruses

Lower respiratory tract infections (LRTIs) are among the leading causes of ICU resource utilization (4). Before the advent of the SARS-CoV-2 pandemic, the incidence of viral-associated LRTIs requiring ICU admission varied greatly among different studies, and ranged between 16 and 49%, with the most frequently isolated viruses being influenza, human rhinoviruses (HRhV), human coronaviruses (HCoV), respiratory syncytial virus (RSV), human metapneumovirus (HMPV), human parainfluenza viruses (HPIV), and adenoviruses (HAdV) (4–7). Common viruses are detected in respiratory samples of patients with nosocomial pneumonia with an incidence ranging from 13.8 to 32% in adults, and as high as 69.2% in pediatric population (8–13).

Current seasonal influenza epidemics are caused by influenza A strains (A/H3N2 and the 2009 pandemic strain H1N1—A/H1N1pdm09) and influenza B lineages (B/Yamagata and B/Victoria), resulting in 3–5 million cases of severe infections annually around the world (14). The annual casualties from influenza epidemics are estimated up to 650,000 people globally, while the respiratory excess mortality rate is estimated between 0.1 and 6.4/100,000 for patients younger than 65 years, 2.9 and 44/100,000 for people 65 and 74 years old, and as high as 17.9–223.5/100,000 for those older than 75 years old (15). The Centers for Disease Control and Prevention (CDC) reported that annual estimates of flu cases, hospitalizations and deaths in the United States (U.S.) ranged among 9–45 million, 140,000–810,000 and 12,000–61,000, respectively during 2010–2020, with the 2017–2018 flu season bearing the highest disease burden (16). A U.S. study estimated that among 114,000–633,000 influenza-hospitalizations during three flu seasons (2010–2013), 18,000–96,000 patents required ICU admission (17). The same study demonstrated that the risk of death during influenza admissions increased with age (0.2–0.9% in children<18 years old, 1.8–2.8% of adults 18–64 years old, and 3.4–4.7% of those ≥65 years old), and the majority of hospitalizations (54–70%) and deaths (71–85%) occurred in patients aged ≥65 years old (17).

**Figure 1:**
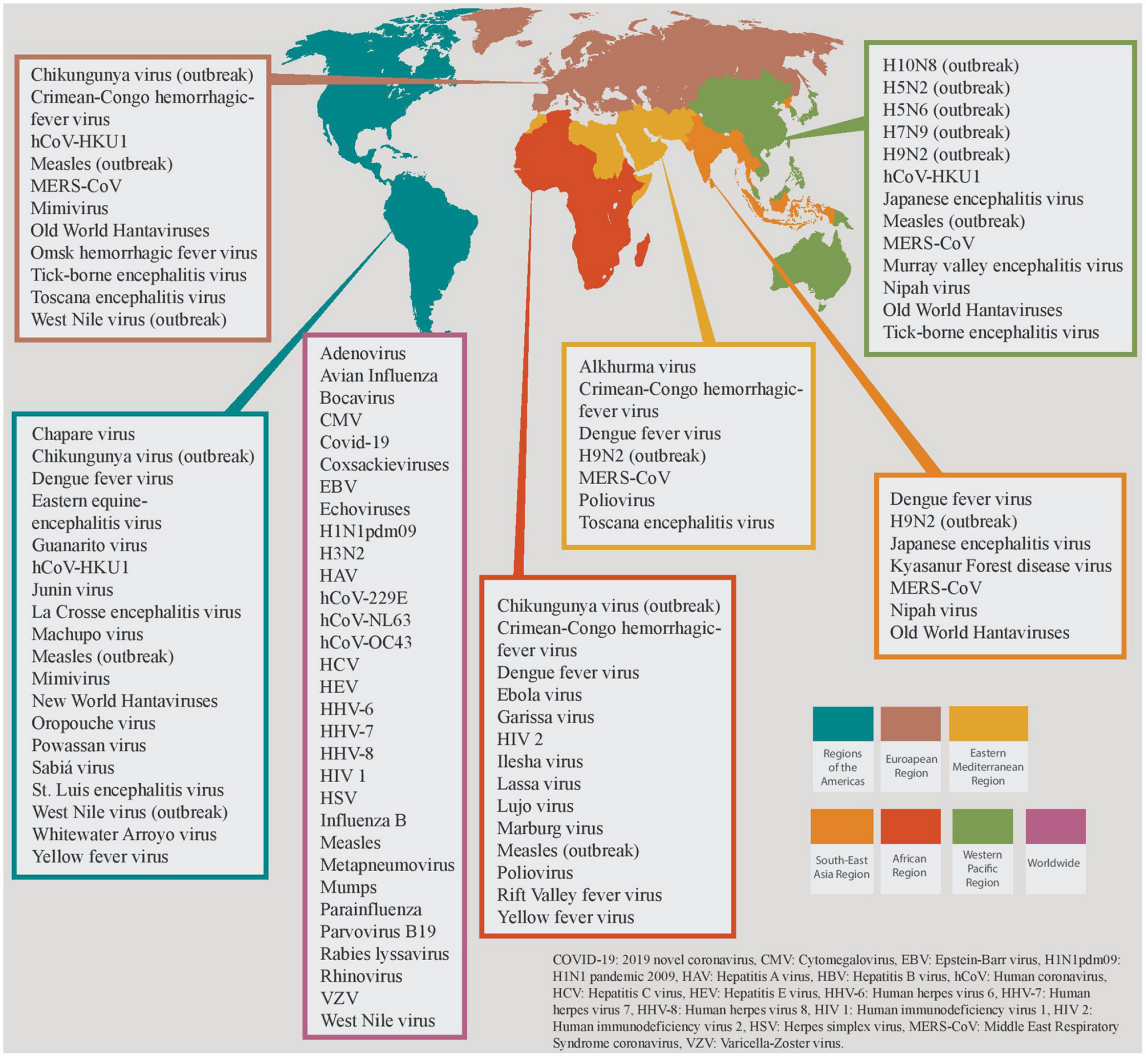
Global distribution of viruses causing critical illness divided in the 6 World Health Organization (WHO) Regions. Viruses that are restricted in specific WHO regions are grouped together, and different colors are attributed to each region. Viruses that share a worldwide distribution are grouped in the magenta colored box.

HCoVs are ubiquitous viruses with worldwide distribution and yearlong transmissibility [Figure 1; (18, 19)]. Among them, three betacoronoviruses have caused epidemics of severe pneumonia within the last 20 years. Severe acute respiratory syndrome coronavirus (SARS-CoV) and Middle East respiratory syndrome coronavirus (MERS-CoV) caused outbreaks and epidemics of severe acute respiratory infections mainly in China and the Arabian peninsula (19–22). SARS-CoV-2 emerged in China in December 2019 and has led to an unprecedented pandemic with more than 90 million confirmed cases of the so-called Coronavirus Disease 2019 (COVID-19) and nearly 2 million deaths across the globe (23). Since the onset of the pandemic, cumulative data from the U.S. demonstrate that the majority of SARS-CoV-2 infections (54%) are diagnosed among people aged 18–49 years old; while only 10% of the total diagnoses are made in older adults (≥65 years old), this age group represents the majority of symptomatic cases and COVID-19-associated hospitalizations (90 and 46%, respectively) (24). Both symptomatic illness and hospitalization rates are very low among children aged 0–4 years old (3 and 1%, respectively) (24). The current global case fatality rate ranges between 1.1 and 4.4% among different countries, although during the first wave of the pandemic this rate was much higher in Italy and Spain, reaching up to 14.5 and 11.4%, respectively (25). Finally, the number of COVID-19 patients requiring ICU admission varies greatly among countries and among the different phases of the pandemic, but it ranges between 0.2 and 108 cases per million population (26). MERS-CoV causes outbreaks in Middle East mainly within hospital settings (27). Since the first confirmed case in 2012, the World Health Organization (WHO) has reported 2,494 cases of MERS-CoV in 27 countries and 858 deaths among them, leading to a case fatality rate of 36% (28, 29).

HPIVs are common causes of RTIs, targeting primarily young children and causing nearly 40% of all acute RTIs in this age group (30). Adults can also be infected; up to 11.5% of adult hospitalizations for RTIs attributed to HPIVs (31–33). An epidemiological study in Kuwait demonstrated that HPIVs were isolated from 2.6% of immunocompetent patients with viral infections requiring admission in pediatric and adult ICUs (34). Concerning HRhV, a recent epidemiological study in mechanically ventilated adult patients in five ICUs demonstrated that HRhV were the second most prevalent virus (5%) after HPIV (6%), independently of the cause of ICU admission (35). Moreover, the prevalence of HRhVs was significantly higher in patients with severe RTIs (9.6%) compared to those admitted for other reasons (2.6%), thus signifying their contribution in severe LRTIs (35). Another retrospective study reported a prevalence of HRhV of 7.5% and a mortality rate of 18.2% among adult critically ill patients, although not all these deaths were directly connected to HRhV detection; however, among all HRhV-positive patients, 59.1% fulfilled the definition of pneumonia (36). The incidence of RSV infections in adults is surprisingly higher than what it was previously believed. Notably, RSV is detected in 12% of all medically attended adults aged ≥50 years old with RTIs (37–39). In 2015, about 1.5 million older adults in industrialized countries suffered from RSV-associated acute RTIs, and 214,000 (14.5%) were admitted to hospital; the same year, RSV was associated with 14,000 in-hospital deaths in older adults globally (40). According to data from the U.S., the annual incidence of RSV infection in elderly adults has been reported about 3–7% and 4–10% of healthy and high-risk patients, respectively (41). Moreover, this study found similar clinical outcomes for both RSV and influenza A infections in terms of length of hospital stay, need for ICU and mortality rates (41). Specific adult groups are at high risk for severe infections and intubation form HMPV infections, but younger patients may also develop severe LRTI and ARDS requiring intubation and mechanical ventilation (35, 42–44). A recent multicenter study in France found an incidence of HMPV-positive patients of 3% among 3,148 hospitalized adults with acute RTIs (45). In this study, 21% required admission in ICU and 4% died during hospitalization among HMPV-positive patients (45). Other studies have reported HMPV-related mortality rates in adults comparable with or slightly lower than those of influenza and RSV (43, 44).

### Other Viruses

The most commonly identified viral pathogens among critically ill patients with neurological infections (mainly encephalitis and meningoencephalitis) include herpes simplex virus (HSV), varicella zoster virus (VZV), and arboviruses like West Nile Virus (WNV). The epidemiology of the viruses that are implicated in central and/or peripheral nervous system infections is outlined in Table 1.

**Table 1:** Epidemiology of main viruses causing neurologic and neuromuscular syndromes.

**Viral pathogen**	**Epidemiology**	**References**
Herpes simplex virus (HSV)	• Most common cause of sporadic viral encephalitis (2–6/1.000.000) • 20% of viral encephalitides • More than 90% HSV-1 • 60% of HSV encephalitis cases require ICU admission • 32.8 and 17% of HSV encephalitis are admitted in ICU and require mechanical ventilation, respectively • Mortality: 10–20%	(46–49)
Varicella zoster virus (VZV)	• Second most common pathogen of sporadic viral encephalitis. • Incidence 5.3/1.000.000 • 4–14% of encephalitides • Mortality: 15% in immunocompetent, nearly 100% in immunocompromised	(46, 50, 51)
Arboviruses	• Viral encephalitis restricted in particular regions • North America: WNV, LCEV • Europe: TBEV, WNV • Among the most common are Dengue, Zika, and WNV • Morbidity and mortality: depends on the type of the pathogen	(3, 46, 52, 53)
Non-polio enteroviruses (EV)	• Mainly in children • Outbreaks of AFP caused by EV-A71 have been reported in Hungary, Bulgaria, India, Taiwan, and Colorado, USA • Outbreak in USA attributed to EV D68 caused AFP, encephalitis and meningitis (54% in ICU, 23% required intubation) • Echoviruses 11, 13, 18, 30 are causes of AFP and encephalitis	(54–58)

*AFP, acute flaccid paralysis; ICU, intensive care unit; LCEV, La Crosse Encephalitis Virus; TBEV, tick-borne encephalitis virus; USA, United States of America; WNV, West Nile Virus*.

Ebola virus is among the deadliest pathogens of viral hemorrhagic fever (VHF) syndrome, with a case fatality rate varying from 25 to 90% and an average mortality rate of 50% (59). Between 2014 and 2016, an Ebola epidemic resulted in more than 28,000 cases with more than 11,300 casualties in Sierra Leone, Liberia and Guinea, while an outbreak is currently ongoing in West Africa [Figure 1; (59)]. Marburg virus has a case fatality rate ranging from 24% to as high as 88% (average 50%) (60). The virus was initially discovered during a European outbreak caused by imported African monkeys in 1967, and since then is responsible for sporadic cases and outbreaks mainly in Central and South Africa (60). Dengue virus is currently endemic in more than 100 countries across 5 WHO regions, and is accountable for approximately 390 million infections annually; although Asia has about 70% of the global dengue burden, a steep worrisome rise in cases and associated deaths was observed in 2019 across several countries (61). The disease has also caused outbreaks in the European continent, representing the second most common cause of fever (after malaria) in returning travelers from low- and middle-income countries, although these data were drawn before the onset of SARS-CoV-2 pandemic (61). Severe dengue requiring hospitalization is estimated to affect 500 000 people annually, with a case fatality rate of 2.5% (62). Lassa virus is currently endemic in Western Africa (63). In contrast to other VHF pathogens, 80% of infected people remain asymptomatic with an overall mortality rate being as low as 1%; however, case fatality rate among those who develop VHF is 15% (63). Of note, pregnant women at the third trimester are at increased risk for severe infection with Lassa virus with high mortality rates (maternal death and/or fetal loss occurring in more than 80% of the cases) (63). Hantaviruses are divided into the “New World” strains, that cause sporadic cases and outbreaks of “hantavirus cardiopulmonary syndrome” (HCPS) mainly in the Americas, and the “Old World” strains that cause the “hemorrhagic fever with renal syndrome” (HFRS) in Europe and Asia (64, 65). HCPS is a potentially fatal disease with a case fatality rate of 40% (66). One third of the HFRS-deaths are associated with fulminant unresponsive shock. However, case fatality rate in HFRS is not as high as the HCPS, which ranges from <1% to 5–15% (67). Finally, the Crimean-Congo hemorrhagic fever (CCHF) virus is distributed in countries of the Africa, the Balkans, the Middle East and the Asia, and causes outbreaks with mortality rates up to 40% (68).

## Pathogenesis of Severe *de novo* Viral Infections and Viral Reactivation

### Physiological Host Responses to Viruses

Adequate innate and adaptive immune responses play a pivotal role in controlling viral infections. Innate immune response begins with the recognition of pathogen-associated molecular patterns (PAMPs) by the pathogen recognition receptors (PRRs) of the host cells (69). The activation of these receptors via signaling pathways induces the production of pro-inflammatory cytokines and anti-viral molecules, notably type I and III interferons (IFNs), as well as the migration of innate immune cells at the site of infection (69). Dendritic cells bridge innate and adaptive immunity by mediating antigen presentation in lymphoid tissues (70). Viral peptides are presented to CD8+ T cells by major histocompatibility complex (MHC) class I molecules, leading to their transformation into effector cells. CD4+ helper T cells are activated by the recognition of antigen-MHC class II complexes on antigen-presenting cells and induce both CD8+ T cell and B cell responses, the latter leading to specific antibody formation (71).

### Factors Associated With Severe Viral Infections and Immune Dysregulation

#### Viral Immune Evasion

Failure of host defense against viral pathogens may be caused either by direct viral-immune evasion or by predisposing host factors. HSV and measles viruses, for example, directly inhibit the maturation of dendritic cells (72). Other viruses, such as Human Immunodeficiency virus (HIV), Hepatitis B virus (HBV) and Hepatitis C virus (HCV), produce anti-inflammatory molecules, especially interleukin-10 (IL-10). Studies have also pointed out the role of PRR inhibition, like retinoic acid-induced gene I (RIG-I) in influenza A, paramyxovirus, and HCV infection (72). Moreover, HSV, Cytomegalovirus (CMV), Epstein Barr virus (EBV), and HIV are able to disrupt the crucial step of antigen presentation. Some viruses, especially those who belong in the *Herpesviridae* family, become latent, and can reactivate later in response to stress or immunosuppression [Table 2; (73)].

**Table 2:** Viruses reactivated in immunocompetent and immunocompromised critically ill patients.

**Viral pathogen**	**Type of infection/disease**	**Clinical implications**
**Stress-induced immunosuppression**		
BK poliomavirus	Detectable in urine	Unknown clinical significance
Cytomegalovirus (CMV)	Detection in blood and/or bronchial secretions	Treatment based on clinical judgment-not universally recommended, unknown clinical significance
Epstein-Barr virus (EBV)	Viremia	Same as above
Herpes simplex virus (HSV)	Detectable in bronchial lavage fluids	Same as above
Human herpesvirus 6 (HHV-6)	Viremia, DRESS	Undetermined role in DRESS pathogenesis
Human herpesvirus 7 (HHV-7)	Viremia	Unknown clinical significance
Human herpesvirus 8 (HHV-8)	Viremia	Same as above
JC poliomavirus	Detectable in urine	Same as above
Varicella-zoster virus (VZV)	Viremia, Herpes zoster	Treatment based on clinical judgment-not universally recommended, unknown clinical significance. May be associated with protracted fever in ICU children
Transfusion transmitted virus (TTV)	Viremia	Unknown clinical significance
**Iatrogenic or HIV-related immunosuppression**		
Human adenovirus (HAdV)	Multiple clinical syndromes	Increases morbidity and mortality
BK poliomavirus	Renal allograft infection (interstitial nephritis), ureteral stenosis, hemorrhagic cystitis. Less common: pneumonitis, retinitis, liver disease, and meningoencephalitis	Progressive graft failure. Appears usually within the first year of transplantation. Management includes immunosuppression reduction (but risk for graft rejection) Cidofovir, leflunomide, and ciprofloxacin have been used in few cases—not enough data
Cytomegalovirus (CMV)	Multiple clinical syndromes	Increases morbidity and mortality, graft rejection, predisposes to other infections
Epstein-Barr virus (EBV)	PTLD	Long-term sequelae of transplantation
Hepatitis B virus (HBV)	Asymptomatic transaminitis to fulminant acute hepatitis	Reactivation can lead to severe acute hepatitis and acute liver failure requiring liver transplantation. Risk for re-infection of the liver graft
Hepatitis C virus (HCV)	Viremia, increased aminotransferases	Usually not clinically important reactivation. Should be treated with DAAs
Human herpesvirus 6 (HHV-6)	Viremia	Unknown clinical significance
Human herpesvirus 7 (HHV-7)	Viremia	Unknown clinical significance
Human herpesvirus 8 (HHV-8)	Kaposi sarcoma	Visceral Kaposi sarcoma may lead to fatal hemorrhage (e.g., hemoptysis, melena, hematemesis)
JC poliomavirus	PML (possibly also involved in numerous neoplasms)	Management includes immunosuppression reduction and ART initiation in HIV/AIDS
Parvovirus B 19	PRCA	Severe refractory anemia
Varicella-zoster virus (VZV)	Viremia, herpes zoster, hepatitis, cholangitis, gastritis, esophagitis, pancreatitis, paralytic ileus, hyponatremia (with or without eruption)	Treatment with aciclovir and prophylaxis with oral valaciclovir

*AIDS, Acquired Immunodeficiency Syndrome; ART, antiretroviral therapy; DAAs, direct-acting antiviral agents; DRESS, drug reaction with eosinophilia and systemic symptoms; HIV, human immunodeficiency virus; ICU, intensive care unit; PML, progressive multifocal leukoencephalopathy; PRCA, pure red cell aplasia; PTLD, post-transplant lymphoproliferative disorder*.

#### Host Factors Associated With Immune Dysregulation

**Figure 2:**
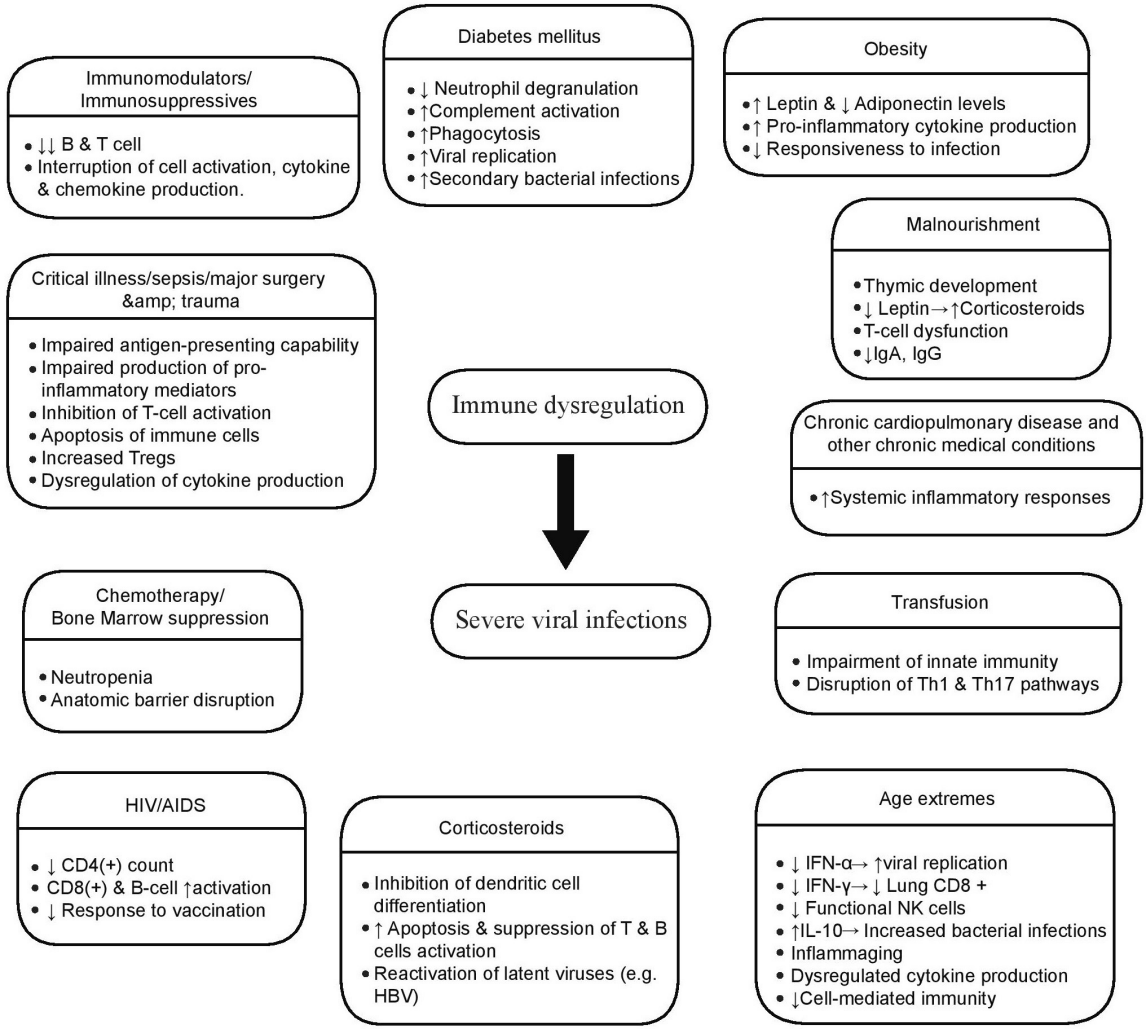
Host factors associated with severe viral infections. Multiple host conditions produce a dysregulated immune state that posits the host in risk of developing a serious viral infection. These factors affect both innate and adaptive immune mechanisms in various ways, the most significant of whom are illustrated here.

Numerous host factors contribute to a dysregulated immune response and augment viral virulence leading to severe viral infections with multi-system involvement [Figure 2; (74–83)]. Iatrogenic immunosuppression, HIV-related and stress-induced immunosuppression, comorbidities such as diabetes mellitus, cardiovascular disease and chronic obstructive pulmonary disease, as well as factors like gene polymorhisms, age extremes, and pregnancy are risk factors for severe viral infections like influenza and COVID-19 (84–86).

Age extremes significantly affect immune responses, and this has an impact on morbidity and mortality of viral infections, such as influenza and SARS-CoV-2 (87). Immature responsiveness of immune system in the first years of life leads to increased risk of severe RSV and influenza infection (88). In old age, serious disturbances in both innate and adaptive immunity lead to dysregulated responses, affecting viral clearance and disease severity, as dramatically seen in COVID-19 disease. Age changes in the immune system include, among others, the impaired production of type I IFNs from dendritic cells, hampered natural killer cell function, reduced responsiveness of T and B cells to antigen, and decreased repertoire of CD8+ T cells, partly due to expansion of inflationary CD8+ T cells caused by chronic viral infection, such as CMV (87). Additionally, inflame-aging, a well-described pro-inflammatory cytokine excess state in old age, contributes to the immune dysregulation (87, 89).

Obesity has also been associated with increased susceptibility to infections leading to high rates of poor outcomes, including influenza and COVID-19 (90, 91). In this patient group, contributing factors are considered to be mechanical, such as increased airway resistance and impaired gas exchange, as well as the underlying immune dysfunction driven by decreased adiponectin production (91). Adiponectin, an adipokine that is reduced in obesity, normally downregulates macrophage activation and pro-inflammatory cytokine production (91). Thus, low adiponectin, high leptin levels, and high leptin-resistance contribute to a heightened pro-inflammatory cytokine production in obese patients, and a subsequent blunted immune response to infection by influenza (83). Low adiponectin levels have been reported during pregnancy as well; however, whether this is a key element of immune dysregulation observed in pregnant women with severe influenza infection, is still not clear (83). Besides baseline host risk factors, other iatrogenic interventions in critically ill patients may contribute to immune dysregulation leading to increased rates of (severe) viral infections, like immunomodulators and blood transfusion. Blood transfusion, especially in the peri-operative period for surgical patients, confers an additional degree of immunosuppression (81). The transfusion of allogeneic blood has known immunosuppressive effects and its impact on the emergence of nosocomial infections, including viremia, viral pneumonia and viral reactivation, has been documented (92–94). The adoption of leukoreduction as an institutional policy for all critically ill patients irrespective of prior immunodeficiency status may be an efficient method in reducing the consequences of transfusion-related immunomodulation.

### Stress-Induced Immunosuppression and Viral Reactivation

After primary infection, multiple viruses fall into latency within the host cells; characteristic examples are the members of *Herpesviridae* family, which become dormant inside neurons (VZV, HSV-1, HSV-2), B cells (EBV), or myeloid cells (CMV). The capacity of latency allows these viruses to reactivate in response to triggering factors, like stress-induced immune paresis leading to clinical manifestations that vary from minor infections to serious complications (Table 2). A prospective cohort study demonstrated that non-immunocompromised patients with sustained sepsis exhibited considerably high frequencies of detectable viral DNA from a variety of viruses like CMV (24.2%), EBV (53.2%), HSV (14.1%), human herpesvirus 6 (HHV-6) (10.4%), and Transfusion transmitted virus (TTV) (77.5%), with two or more viruses being detected in 42.7% of these patients (95).

CMV infection is defined by the isolation of CMV antigens or nucleic acid in body fluids or tissue specimens, as opposed to CMV disease that necessitates the presence of concurrent symptoms and/or signs (96). According to multiple meta-analyses, the incidence of CMV infection in non-immunocompromised critically ill patients has been reported as high as 35%, occurring usually between the 14th and 21st day of ICU admission (97, 98). The highest incidence is observed among patients with prior seropositivity, indicating that reactivation rather than primary infection is the main mode of CMV infection in this population (98). The flawed balance between exaggerated inflammation and transient immunosuppression in the subset of patients with sepsis sets the grounds for CMV reactivation. Tumor necrosis factor alpha (TNF-α) promotes directly CMV gene expression (99). As T cell immunity plays a major role in suppressing the CMV replication, both CMV-induced immunodeficiency and sepsis-induced immune dysfunction may contribute in CMV reactivation (100–102). A meta-analysis by Lachance et al. reported that CMV reactivation in immunocompetent critically ill patients is associated with adverse outcomes, including higher ICU and overall mortality, prolonged duration of mechanical ventilation and ICU stay, more nosocomial infections and increased need for renal replacement therapy (103).

HSV reactivation has been frequently reported in non-immunocompromised critically ill patients (104). Isolation of HSV from bronchoalveolar lavage fluid specimens has been associated with adverse outcomes, like longer duration of mechanical ventilation, prolonged ICU stay and increased mortality (105). HSV detection has been associated with several risk factors in critically ill patients, such as intubation, prolonged mechanical ventilation, ARDS, extensive burns, advanced age, HSV-IgG seropositivity, use of corticosteroids and herpetic mucocutaneous lesions (97). However, it remains unclear whether HSV isolation in mechanically ventilated patients equals to actual HSV infection (either as reactivation or as a *de novo* infection) or it is just a surrogate marker of underlying disease severity (106). Hence, it is still controversial whether, how or when treatment should be applied in these cases (107, 108).

VZV reactivation has been associated with prolonged duration of fever in immunocompetent children in the ICU (109). EBV DNA, detected in blood samples of ICU patients, has been correlated with increased morbidity and mortality (110). Nonetheless, the clinical significance and the need of pre-emptive treatment of these reactivations remain obscure and will only be answered by large, well-designed, randomized controlled trials in the diverse population of immunocompetent critically ill patients.

### Immunocompromised Patients and Viral Reactivation

The advent of potent immunosuppressive drugs has increased the survival rate of previously bleak diagnoses, especially in hematological patients and solid organ transplant recipients (SOTRs), but at the same time has increased the incidence of opportunistic viral infections and viral reactivations leading to an upsurge in the need for higher level of care.

#### Hematopoietic Stem Cell Transplant Recipients and Neutropenic Patients

Viral reactivation in hematopoietic stem-cell transplant recipients (HSCTRs) is associated with significant morbidity and mortality. Pre-transplantation evaluation guidelines have been designed to prevent post-transplant infections. Seropositivity screening for CMV, HBV, HCV, VZV, HSV, EBV, and HIV in HSCT donors or candidates outlines the infection control policies and prophylactic regimens necessitated in the post-transplant period (111, 112). According to published guidelines (111) for the prevention of HSV, VZV, and CMV re-activation, seropositive HSV HSCTRs should receive antiviral prophylaxis with intravenous aciclovir from conditioning until engraftment or until mucositis resolves, seropositive VZV HSCTRs should receive antiviral prophylaxis for at least 1 year, preferably with oral valaciclovir while CMV seropositive HSCTRs of allografts from seronegative or seropositive donors, and seronegative HSCTRs of allografts from seropositive donors should be monitored weekly with quantitative CMV testing. Pre-emptive treatment should be started in these patients when there is detectable CMV viremia with intravenous ganciclovir or oral valganciclovir.

Apart from the HSC transplantation, CMV reactivation has been reported in the setting of febrile neutropenia in hematologic patients (113). Neutropenia, especially when the count is <500 cells/μL, increases the risk of opportunistic infections including viral infections or reactivations. Therefore, in episodes of febrile neutropenia in patients with hematologic malignancies, viral *de-novo* infections or reactivations should always be considered (114, 115).

#### Solid Organ Transplant Recipients

Infections remain a major impediment in SOTRs; as for HSCTRs, laboratory testing is undertaken in the pre-transplant period to evaluate for past infectious exposures and exclude active infections with serology test for CMV, HSV, VZV, EBV, HIV, HBV, and HCV (116). The highest incidence of viral infections occurs in the subset of immunologically naïve patients who receive grafts from infected donors.

Viral reactivation in SOTRs leads to a wide array of clinical manifestations. Examples include neutropenic fever, colitis, adrenal insufficiency, meningoencephalitis (CMV), cholangitis (VZV), and encephalitis (HSV, JC virus). Apart from specific clinical syndromes, viral reactivation has collateral immune effects including further immunosuppression, that predisposes to the emergence of other opportunistic infections as well as an upsurge of cancer risk (117–120). Improvement in graft function and inhibition of allograft rejection by CMV prophylaxis and antiviral treatment provides evidence for a possible CMV implication (121). Although the exact immunologic mechanisms of this process remain unknown, it has been suggested that heterologous immunity via CMV-specific T cells producing proinflammatory cytokines as well as MHC class II and adhesion molecules upregulation leading to increased alloimmunity, may explain the association between CMV infection and graft rejection (122–124). Implemented strategies for the prevention of viral reactivation in this population include universal prophylaxis with valganciclovir or ganciclovir for patients at risk for CMV reactivation (seropositive recipients or recipients of seropositive donors) (125, 126). The type of the organ transplantation and the institutional policies determine the duration of therapy. However, late CMV infection can be manifested after discontinuation of prophylaxis (127, 128). Extending prophylaxis or a pre-emptive approach with viral load monitoring are possible ways to overcome this problem (115, 129). Patients not on CMV prophylaxis should receive HSV and VZV prophylaxis for the first 3–6 months and at any point of immunosuppression increments. Since no effective antiviral therapy exists for EBV, monitoring of high-risk patients (e.g., seronegative recipients) at predetermined intervals is the approach of choice.

#### Human Immunodeficiency Virus Associated Viral Reactivations

Members of the *Herpesviridae* family, including CMV, EBV, and HSV, may reactivate causing various clinical syndromes in people living with HIV (PLWH). The presence of positive heterophile antibody tests in the setting of acute retroviral syndrome suggests a possible reactivation of EBV (130). Late presenters with a CD4+ count <50 cells/μL are at high risk of CMV-related disease, more commonly retinitis or encephalitis (131, 132). HSV co-infected patients exhibit increased frequency of HSV reactivation, mostly with atypical symptoms (133, 134). Unusual manifestations include esophagitis, tracheitis, meningoencephalitis, hepatitis, pneumonitis, retinal necrosis and disseminated disease (135). Reactivation of HSV in PLWH alters the natural history of HIV infection, with more efficient sexual transmission and increased rate of viral replication, during clinical or subclinical HSV reactivation (136). JC poliomavirus is implicated in the development of progressive multifocal leukoencephalopathy (PML) and possibly in various human neoplasms, in people with Acquired Immune Deficiency Syndrome (AIDS) or other severe immunosuppressive status (137).

#### Hepatitis B Virus Reactivation in Immunosuppressed Patients

After HBV infection, the viral DNA remains in hepatic cells of all patients irrespectively of serological recovery. The host's immune response plays a vital role in controlling viral replication (138). Critically ill patients with a history of HBV infection that are going to receive aggressive immunosuppression for their underlying diseases are at risk of HBV reactivation with grievous outcomes. Screening for HBV infection with HBV surface antigen (HBsAg) and HBV core antibody (anti-HBc) must be obtained before the initiation of immunosuppression therapy (including prednisone monotherapy ≥20 mg/day for more than 4 weeks) (139–141). The patients are risk-stratified based on the status of HBsAg and the type of immunosuppression (141). HBV reactivation may range from asymptomatic, with the only sign being a rise in HBV DNA level, to overt clinical syndrome with deranged aminotransferases and/or fulminant hepatitis (139).

#### Other Viral Pathogens Reactivated in Immunosuppressed Patients

Adenoviruses can exhibit a variety of clinical syndromes in immunocompromised hosts (142). The concept of adenoviral persistence leading to a subclinical state of infection has only recently been reported (143). *In vitro* studies have shown that reduced interferon levels promote viral reactivation (144). There is sufficient evidence showing that viral reactivation of endogenous adenoviruses is the major route of infection acquisition in HSCTRs and SOTRs (145). HSCTRs can present with a wide array of organ-specific adenoviral disease, including pneumonia, colitis, hepatitis, hemorrhagic cystitis, encephalitis, and disseminated disease (146). Adenoviral disease in SOTRs involves more commonly the donor organ, thus generating organ-related clinical manifestations (147). Since few data are available for effective antiviral agents against adenoviruses, contact, and droplet precautions remain the mainstay of infection control in the population of critical care transplanted patients (111).

## Diagnosis

### Pathogen Detection Techniques

Diagnosis of viral infections cannot be solely based on symptoms and signs, especially among critically ill patients who may have atypical clinical presentations. Laboratory-based techniques like viral cultures, electron microscopy and serology have been largely substituted by nucleic acid amplification tests (NAATs), sequencing (including next generation sequencing) and antigen detection methods, due to their increased accuracy and faster turnaround times (148, 149). Many commercially available rapid tests using nucleic acid amplification or antigen-based techniques are currently available for respiratory viruses (148, 150, 151). NAATs have advanced the sensitivity and specificity of influenza detection, as well as the identification of mutant variants that may be associated with increased disease severity, like the NA V263I and NS1 K196E for the influenza A H3N2 strain and the D222G for the influenza H1N1 pandemic strain 2009 (1, 152–154). Similarly, the diagnosis of SARS-CoV-2 infection is based on real time reverse transcription polymerase chain reaction (rRT-PCR) in respiratory tract specimens, although sensitivity of rRT-PCR is not 100% (155). Many factors may jeopardize the accuracy of the currently utilized SARS-CoV-2 NAATs, like inappropriate specimen collection techniques, handling of specimens, presence of interfering substances or antiviral treatment, manual errors, sample contamination, and testing outside the diagnostic window; all these factors should be considered when interpreting a negative result, especially during an ongoing pandemic (156). Although novel techniques have advanced our capacity to detect viruses, one should keep in mind that detection does not always correlate with pathogenicity (157). Results of such tests should guide clinical decisions in the context of a compatible clinical syndrome. Although antigen-based diagnostics for SARS-CoV-2 are generally less sensitive than molecular techniques, their utilization is rising globally, as they are usually less expensive than NAATs and provide results within minutes of sample acquisition (158).

Diagnosis of central nervous system (CNS) infections is typically made by detecting the viral genome in cerebrospinal fluid (CSF) by PCR, which yields high sensitivity and specificity rates (159). False negative results may occur early in the course of the infection (<72 h), in which case it is prudent to repeat PCR a few days later (159). Intrathecal serology may complement nucleic acid detection in some viruses like in VZV and arboviral CNS infection (159, 160).

### Imaging Modalities

Imaging studies in viral pneumonias have low specificity compared to molecular tests, but can complement and guide both diagnosis and management of critically ill patients. Distinct patterns of chest computed tomography (CT) scan, may distinguish between some viral families that tend to induce specific radiological patterns (161). For SARS-CoV-2-induced pneumonia specifically, chest CT was found sensitive and moderately specific for the diagnosis of COVID-19 in patients with suspected infection, thus offering limited capacity of distinguishing between SARS-CoV-2 infection and other causes of acute lung injury (162).

Brain imaging may be highly characteristic in certain viral CNS infections. High signal on T2-weighted and fluid-attenuated inversion recovery (FLAIR) sequences in temporal lobes, especially when asymmetrical, are highly characteristic magnetic resonance imaging (MRI) findings of HSV encephalitis (163). MRI findings in arboviruses are not specific, but may show a predilection for deep gray matter (basal ganglia) and the brainstem (160).

## Viral Clinical Syndromes in Critically Ill

### Respiratory Infections

Clinical manifestations of viruses causing severe respiratory infections may range from asymptomatic or mild upper RTI to severe pneumonia with respiratory failure, septic shock-like syndrome, multi-organ dysfunction, and numerous other complications (Table 3), depending on both viral and host factors. The WHO has designated the terms of influenza-like illness (ILI) and severe acute respiratory infection (SARI) to embrace the typical presentation of viral pneumonias (188). Since there are no major differences in the initial management of most viral pneumonias (especially those who not have a specific treatment) these umbrella-terms allow healthcare professionals to quickly identify and manage these patients accordingly.

**Table 3:** Clinical manifestations and complications of viruses causing severe respiratory infections.

**Viral pathogen**	**Clinical manifestations**	**Complications**	**References**
Human adenovirus (HAdV)	From mild URTI to (rarely) severe pneumonia, keratoconjunctivitis, gastrointestinal symptoms	• Severe pneumonia/ARDS • Viremia • Severe disseminated disease in immunocompromised • Reports of: hemorrhagic cystitis, fatal myocarditis, cardiomyopathy, pancreatitis, encephalitis, meningitis, mononucleosis-like syndromes, pulmonary dysplasia, hepatitis, nephritis, hemorrhagic colitis, intestinal intussusception in children, and sudden infant death	(146, 164–171)
Human parainfluenza virus (HPIV)	From mild URTI and laryngotracheobronchitis (croup) to bronchiolitis and (rarely) severe pneumonia	• Severe pneumonia/ARDS • COPD and asthma exacerbation • Extrapulmonary complications (parotitis, apnea, bradycardia, exacerbation of nephrotic disease, hepatitis, fatal rhabdomyolysis, febrile seizures, ventriculitis and encephalitis, pericarditis, and myocarditis)	(34, 172, 173)
Influenza	From asymptomatic to severe viral pneumonia. Fever, headache, myalgia, malaise, acute respiratory illness (non-productive cough, sore throat, nasal discharge)	• Primary pneumonia • Bacterial and fungal superinfections • ARDS and influenza-induced sepsis syndrome • Myositis, rhabdomyolysis • Myocarditis, pericarditis, myocardial ischemia • Encephalitis, acute disseminated encephalomyelitis, transverse myelitis, aseptic meningitis, Guillain-Barre syndrome	(84, 174)
MERS-CoV	Severe pneumonia, gastrointestinal symptoms	• Acute kidney failure • ARDS • Pericarditis • Disseminated intravascular coagulation • Profuse diarrhea	(28, 175, 176)
Respiratory syncytial virus (RSV)	From asymptomatic or mild upper URTI to bronchiolitis, severe pneumonia	• ARDS • COPD and asthma exacerbation • Extrapulmonary complications in children (central apneas, seizures, lethargy, feeding or swallowing difficulties, tone abnormalities, strabism, CSF/EEG abnormalities, hyponatremia, hepatitis) • Childhood asthma	(177–180)
SARS-CoV	Fever, malaise, headache, myalgia, non-productive cough, dyspnea	• Respiratory failure/ARDS	(181)
SARS-CoV-2	From asymptomatic to severe pneumonia/respiratory failure and multi-organ dysfunction/failure. Fever, cough, dyspnea, myalgia, diarrhea, anosmia/ageusia, headache, non-productive cough, sore throat, nasal discharge	• ARDS • Arrhythmias, acute cardiac injury, shock • Thromboembolic complications • Cytokine storm syndrome • Multisystem inflammatory syndrome in children • Bacterial and fungal superinfections • Neurological complications (polyneuropathy, myositis, cerebrovascular diseases, encephalitis and encephalopathy, Guillain-Barre syndrome) • Long-term hyposmia	(182–187)

*ARDS, acute respiratory distress syndrome; COPD, chronic obstructive pulmonary disease; CSF, cerebrospinal fluid; EEG, electroencephalogram; MERS-CoV, middle east respiratory syndrome-related coronavirus; SARS-CoV-2, severe acute respiratory syndrome-related coronavirus 2; SARS-CoV, severe acute respiratory syndrome-related coronavirus; URTI, upper respiratory tract infection*.

### Central and Peripheral Nervous System Infections

A wide array of viral pathogens causes encephalitis (Table 4). In affected patients, a detailed travel, exposure and sexual history should be obtained, along with meticulous clinical examination for rashes and/or focal neurologic deficits that could suggest a certain viral pathogen (e.g., HSV). Geographic and epidemiologic clues are frequently the only suggestive features of a specific viral pathogen (Figure 1 and Table 1). Examples include current mosquito activity for arboviral encephalitis, regions with high tick population for tick-borne encephalitides and animal bite wounds for rabies. HSV-1 is the most common cause of sporadic viral encephalitis (189). Prompt and accurate diagnosis of HSV CNS infection is crucial as the timely initiation of targeted treatment has a significant impact on the outcome.

**Table 4:** Neurologic syndromes caused by viral pathogens.

**Viral pathogen**	**Neurologic syndrome**			
	**Meningitis**	**Encephalitis**	**AFP**	**Other**
Adenovirus	**√**	**√**		
BK poliomavirus	**√**	**√**		
CHINV	**√**	**√**		
CMV		**√**		Transverse myelitis, polyradiculomyelitis, ependymitis
Colorado tick fever	**√**	**√**		
Coxsackie A24			**√**	
DENV			**√**	Deep gray matter encephalitis
EBV		**√**		Cerebellitis, CNS lymphoma
Echo 13	**√**		**√**	
Echo 18		**√**		
Echo 30	**√**	**√**		
EEEV	**√**	**√**		
EV A71		**√**	**√**	
EV D68			**√**	
HHV-6				HHV-6 PALE
HSV-1		**√**		
HSV-2	**√**			
Influenza (A & B)	**√**	**√**		Transverse myelitis, Acute necrotizing encephalopathy
JC poliomavirus				PML
JEV			**√**	Deep gray matter encephalitis
La Crosse encephalitis virus	**√**	**√**		Hydrocephalus
Measles		**√**		Acute post-infectious encephalitis Inclusion body encephalitis Subacute sclerosing panencephalitis
Metapneumovirus		**√**		
MVEV			**√**	Deep gray matter encephalitis
Nipah virus		**√**		Polyneuritis cranialis, cerebellitis
Parainfluenza virus		**√**		Ependymitis
Poliovirus/cVDPV		**√**	**√**	Polyneuritis cranialis, VAPP
Powassa virus	**√**	**√**		
Rabies Lyssavirus		**√**	**√**	
Rift Valley Fever virus	**√**	**√**		
SLEV			**√**	Deep gray matter encephalitis
TBEV			**√**	Deep gray matter encephalitis
Toscana virus	**√**	**√**		Hydrocephalus
VEEV	**√**	**√**		
VZV	**√**	**√**		Polyneuritis cranialis, transverse myelitis, cerebellitis, arteritis, vasculopathy
WEEV	**√**	**√**		
WNV			**√**	Deep gray matter encephalitis
ZIKAV			**√**	Deep gray matter encephalitis

*AFP, acute flaccid paralysis; CHINV, Chikungunya Virus; CMV, cytomegalovirus; CNS, central nervous system; cVDPV, circulating vaccine-derived poliovirus; DENV, Dengue Virus; EBV, Epstein-Barr Virus; EEEV, east equine encephalitis virus; EV, enterovirus; HHV-6, Human Herpes Virus-6; HSV, Herpes Simplex Virus; JEV, Japanese Encephalitis Virus; MVEV, Murray Valley Encephalitis Virus; PALE, post-transplantation acute limbic encephalitis; SLEV, St. Louis Encephalitis Virus; TBEV, tick-borne encephalitis virus; VAPP, vaccine-associated paralytic polio; VEEV, Venezuelan Equine Encephalitis Virus; VZV, Varicella Zoster Virus; WEEV, west equine encephalitis virus; WNV, West Nile Virus; ZIKAV, Zika Virus*.

Fever, headache, lethargy and clinical signs of meningismus are features of aseptic meningitis. Abnormal sensorium is the hallmark of encephalitis, and although not often, signs of both entities can be found concurrently leading to the diagnosis of meningoencephalitis. Viral encephalitis and meningoencephalitis frequently lead to altered mental status and decreased level of consciousness that warrants intubation and management in ICU. Acute disseminated encephalomyelitis (ADEM) is a post-infectious sequela of viral encephalitis, precipitated by numerous possible viruses that may necessitate the need of critical care due to coma, seizures, or tetraplegia (190). The absence of viral detection along with histological findings of perivascular inflammation and demyelination suggest an immune-mediated disease (191). Other viral illnesses, not necessarily confined in the brain, can produce post-viral neurologic syndromes with prominent clinical manifestation from the CNS leading to severe morbidity and mortality (Table 5).

**Table 5:** Post-viral neurologic syndromes.

**Syndrome**	**Viral causes**	**Clinical features**	**Treatment**
Acute Disseminated Encephalomyelitis (ADEM)	*Viruses*: Smallpox, VZV, Mumps, Influenza, HIV, HAV, HBV, Herpesviruses, Coxsackie, Coronavirus *Vaccines*: Rabies, Poliomyelitis, Smallpox, JPE, HBV, Influenza, Yellow fever	Rapidly progressive monophasic course (4–5 days) Encephalopathy, ataxia, hemiplegia, hemiparesthesias, cranial nerve palsies, visual changes, seizures (especially children), respiratory failure	Methylprednisolone pulses (3–5 days) followed by oral prednisone (2–6 weeks tapering) IVIG 2 mg/kg for 2–5 days Plasmapheresis
Guillain-Barre Syndrome (GBS)	Herpesviruses, Influenza, Hantavirus, HBV, HEV Rare: WNV, Parvo B19, Rubella, Dengue, Chikungunya, Zika	Rapidly progressive bilateral weakness (ascending flaccid paralysis) Cranial nerve involvement (Miller Fisher syndrome) Sensory disturbances, ataxia, autonomic dysfunction Respiratory failure	Plasma exchange (5 sessions over 2 weeks) IVIG 2 gr/Kg in 2 days Eculizumab (under investigation)
NMDAR encephalitis	HSV, VZV, HHV-6, Enteroviruses	Choreoathetosis (children) Cognitive/behavioral changes (adults) Seizures Autonomic dysfunction Coma	First line: corticosteroids, IVIG, plasma exchange Second line: rituximab, cyclophosphamide
Reyes syndrome	Common: influenza, VZV Rare: coxsackie, parainfluenza, EBV, CMV, adenovirus, hepatitis Associated with aspirin use	Rapidly progressive hepatic failure and encephalopathy	Supportive: treatment of high ICP, seizures and coagulopathy

*CMV, Cytomegalovirus; EBV, Epstein-Barr Virus; HAV, Hepatitis A Virus; HBV, Hepatitis B Virus; HEV, Hepatitis E Virus; HHV-6, Human Herpesvirus−6; HIV, Human Immunodeficiency Virus; HSV, Herpes Simplex Virus; ICP, intracranial pressure; IVIG, intravenous immunoglobulin; JPE, Japanese Encephalitis Virus; NMDAR, N-methyl-D-aspartate receptor; VZV, Varicella Zoster Virus; WNV, West Nile Virus*.

### Shock and Multi-Organ Failure

#### Viral Hemorrhagic Fever

VHF is a group of syndromes that involve fever and hemorrhage of varying severity, caused by four distinct families of RNA viruses: the *Arenaviridae* family, the *Bunyaviridae* family, the *Filoviridae* family, and the *Flaviviridae* family [Table 6; (192)]. Each virus within these families has a distinct geographical distribution as shown in Figure 1. VHF represents the most severe and often fatal form of the infection by these pathogens, with a variable frequency among other clinical syndromes (192). It is characterized by fever, malaise, increased vascular permeability, reduced intravascular volume, coagulopathy and hemorrhagic features (193, 194). As these viruses do not use the same replication strategies inside the hosts, it is unclear how they concur to similar clinical syndromes (195). However, the brisk induction of pro-inflammatory cytokine expression and the fluid distribution imbalance due to (micro)vascular endothelium injury leading to capillary leak and disseminated intravascular coagulation seem to be common pathways leading to VHF development and often to multi-organ failure and death for most of the culprit viruses (195–197).

**Table 6:** Viruses causing different types of shock and multi-organ injury.

**Type of disease**	**Viral pathogens**
**Distributive shock**	
Adrenal insufficiency	Human immunodeficiency virus-1, Cytomegalovirus
Direct and pro-inflammatory cytokine induced endothelial damage	Viral hemorrhagic fever pathogens: ***Arenaviridae*** (Lassa virus, Lujo virus, Junin virus, Machupo virus, Sabiá virus, Chapare virus, Guanarito virus, and Whitewater Arroyo virus) ***Bunyaviridae*** (Hantavirus—HFRS, Crimean-Congo hemorrhagic fever virus, Garissa virus, Ilesha virus, Rift Valley fever virus) ***Filoviridae*** (Ebola virus, Marburg virus) ***Flaviviridae*** (Dengue virus, Yellow fever virus, Omsk hemorrhagic fever virus, Kyasanur Forest disease virus, Alkhurma virus) Other viruses: SARS-CoV-2, Influenza
Dysautonomia	Human immunodeficiency virus, Human T-lymphotropic virus, Herpes viruses, Flaviviruses, Enterovirus 71, Rabies lyssavirus, Varicella zoster (reactivation in autonomic ganglia), Tick-borne encephalitis virus (and viral causes of GBS, myelitis, acute flaccid paralysis)
Pancreatitis	Mumps (the most common), Coxsackie B, Cytomegalovirus, Varicella-zoster virus, Herpes simplex virus, Epstein-Barr virus, Influenza A, Parainfluenza virus, Adenovirus, Measles, Human immunodeficiency virus-1, Hepatitis A and E viruses
Sepsis	**Bacterial superinfections**: Most viral infections predispose to bacterial superinfections and sepsis (most commonly influenza) **Viral sepsis-like syndrome**: Influenza, Viral hemorrhagic fever pathogens (as above), SARS-CoV-2
Viral induced acute liver failure	**Common**: Hepatitis A virus, Hepatitis B virus, Hepatitis E virus **Rare**: Cytomegalovirus, Varicella-zoster virus, Herpes simplex virus, Adenovirus, Epstein-Barr virus, and Dengue virus
**Cardiogenic shock**	
Myocardial ischemia	Influenza A and B, SARS-CoV-2 (e.g., in multisystem inflammatory syndrome in children)
Myocarditis	**Common**: Enteroviruses (mainly coxsackie B) **Less common**: Adenoviruses (fatal myocarditis and cardiomyopathy), Epstein-Barr virus, Hepatitis C virus, Hepatitis E virus, Human herpes virus 6, Human immunodeficiency virus-1, Influenza A, Parvovirus B19, cardiogenic shock due to HCPS (New World Hantaviruses) **Rare**: BK virus, Parainfluenza (HPIV-3), SARS-CoV-2
Pericarditis	Enteroviruses (especially Coxsackieviruses and echoviruses), Herpesviruses (especially Epstein-Barr virus, Cytomegalovirus and Human herpes virus 6), denoviruses, Parvovirus B19, Parainfluenza (HPIV-3)
**Hypovolemic shock**	
Extracellular fluid loss	Cytomegalovirus (colitis), Rotavirus, Norovirus, Enteric adenoviruses (HAdV40 and HAdV41), Astrovirus, Sapovirus
Hemorrhage	Viral hemorrhagic fever pathogens (as above), Adenovirus (hemorrhagic cystitis and hemorrhagic colitis), BK virus (hemorrhagic cystitis), Human herpes virus 8 (visceral Kaposi sarcoma leads to hemoptysis, hematemesis, melena, etc.)
**Obstructive shock (rare)**	
Pulmonary embolism	Influenza (may be associated with pro-coagulant –conflicting data), SARS-CoV-2
Right-sided acute heart failure	Same pathogens as for myocarditis and pericarditis

*GBS, Guillain-Barre Syndrome; HCPS, Hantavirus Cardiopulmonary Syndrome, SARS-CoV-2, severe acute respiratory syndrome coronavirus 2*.

#### Hyperacute and Acute Liver Failure Due to Viral Hepatitis

Acute liver failure (ALF) is defined by the European Association for the Study of the Liver as a rare syndrome characterized by liver injury (elevated serum aminotransferases 2–3 times above the upper limit of normal), impaired liver function (jaundice and coagulopathy with international normalized ratio of >1.5) and hepatic encephalopathy developing within 7 days for hyperacute liver failure (HALF) and 8–28 days for ALF, in patients without pre-existent liver disease (198). Hepatitis E virus (HEV), hepatitis HBV and hepatitis A virus (HAV) are the most common viral pathogens of HALF/ALF, whereas other rare viral causes include CMV, VZV, HSV, and Dengue virus (Table 6) (198). The mortality rate of HEV-induced HALF is generally low, but specific HEV genotypes and patient groups (like the immunocompromised, the elderly, the pregnant women and those with underlying chronic liver diseases) are associated with significant mortality (198, 199). HAV may cause hyperacute ALF, but generally in <1% of the infected, with elderly being at higher risk for HAV-induced HALF and poor survival (200). ALF is a life-threatening condition caused by distributive shock due to generalized vasodilation; electrolyte and metabolic imbalance along with organ hypoperfusion lead to multi-organ dysfunction (198). Therefore, treatment requires admission to critical care settings, close monitoring, tight fluid management, neuroprotective measures, and preparedness for emergency transplantation (198).

## Treatment

### General Preventive Measures

Infection control measures are of paramount importance for prevention of viral infection transmission, especially within hospital settings. Modes of viral transmission can be either direct or indirect depending on the viral strain: direct transmission occurs through contact with infectious materials such as skin, secretions, blood, body fluids, contaminated surfaces and materials, etc. as well as via large droplets (diameter >5 μm), while indirect modes involve transmission through suspended air particles (airborne transmission of droplet nuclei, <5 μm) (201). Typically, droplet and contact precaution measures (for example in influenza and SARS-CoV-2 cases) involve patient isolation, source control (e.g., placement of face mask in non-intubated patients) and donning of level two personal protective equipment (PPE) (202, 203). Patients with airborne viruses such as measles and VZV require isolation in a specialized airborne infection isolation room and source control (as above); furthermore, donning of a level 3 PPE is prerequisite for contacting patients infected with airborne viruses, as well as when aerosol producing procedures are performed in patients with other respiratory viruses like influenza, SARS-CoV-2, and MERS-CoV (202). Ebola and other VHF pathogens are contracted via direct contact with patient's body fluids, contaminated medical supplies and equipment, or contaminated environmental surfaces (204). Therefore, although most blood-borne and contact-transmitted viruses do not require high level self-protective measures, donning of level 3 or higher PPE is essential when caring for patients with Ebola or other human-to-human transmitted VHF viruses (205). Imperatively, proper donning and doffing of PPE as well as meticulous hand hygiene are of paramount importance for infection control and reduction of in-hospital/in-ICU viral transmissions.

### Treatment of CMV and HSV Reactivations in Immunocompetent Critically Ill

Although severe *de novo* viral infections necessitating ICU admission or viral reactivations in immunocompromised patients generally require treatment, provided that an effective therapy or management plan actually exists (Table 7), data on when and how to treat immunocompetent critically ill patients with viral reactivation are still inconclusive. To date, there is no evidence-based guidance for CMV or HSV reactivation treatment in critically ill immunocompetent patients. Regarding HSV reactivation (especially when it is clinically evident, e.g., in the oropharynx), treatment with aciclovir has been proposed in immunocompromised patients, not only because of cytotoxic therapies but also due to conditions such as extensive burns; moreover, treatment should probably be administered in patients with positive HSV test in respiratory samples who have evidence of pulmonary invasion or clinical deterioration that cannot be attributed to other etiology (206, 207). Besides clinical features and organ involvement-directed decision making, it has been proposed that patients with high viral loads (≥10^5^ copies/mL) may also be entitled to treatment for HSV reactivation (208).

**Table 7:** Treatment and prevention of viruses causing severe infection.

**Viral pathogen**	**Antivirals**	**Other treatment**	**Prevention**
Avian Influenza (A/H5, A/H6, A/H7, A/H9, A/H10)	NAIs (oseltamivir, zanamivir)		Infection control, contact precautions with infected poultry
Coxsackie virus A24, Echo virus 13	None	Early intensive physical therapy (for AFP)	None
Crimean-Congo hemorrhagic fever virus	Oral or IV ribavirin	Supportive care	Post-exposure prophylaxis with ribavirin
Cytomegalovirus (CMV)	Immunocompromized: IV ganciclovir, oral valganciclovir (foscarnet if suspected ganciclovir resistance)	Immunocompetent: Supportive care Immunocompromised: CMV immune globulin	For immunocompromized hosts: viral load monitoring, pre-emptive treatment and secondary prophylaxis
Dengue virus, Japanese encephalitis virus	Ribavirin	Mainly supportive care Minocycline, IFNα, IVIG, Plasma exchange have been used, but no clinical trials	Avoid mosquito bites, insecticides Live attenuated tetravalent vaccine for Dengue virus (Dengvaxia®, Sanofi Pasteur Inc.) approved only for seropositive individuals (9–16 years old) in endemic areas Vaccine for Japanese encephalitis virus
Ebola virus	Redmesivir (investigational trials)	mAb114, REGN-EB3 (investigational trials)	Avoid infected animals, infection control
Echo viruses: 18, 30, EV A71, EV D68	Pleconaril (for severe enteroviral infections)		None
Epstein-Barr virus (EBV)	None	Supportive care, corticosteroids for impending airway obstruction	None
Herpes simplex virus 1,2 (HSV-1, 2)	Nucleoside analogs (acyclovir, valacyclovir, famciclovir)	Corticosteroids for encephalitis with severe brain edema	Sex education, chronic suppressive therapy
Human adenoviruses (HAdVs)	Cidofovir (and the pro-drug brincidofovir)	Supportive care, pooled IVIG for immunocompromized patients with severe disease	Infection control, Live oral vaccine for HAdV type 4&7 (available only for some closed populations, e.g., military)
MERS-CoV and SARS-CoV^*^	Lopinavir/ritonavir, IFNa, remdesivir (ribavirin is being tested for MERS-CoV)	Supportive care, Convalescent plasma, immunomodulators, cell therapies, corticosteroids	Infection control Vaccines are under development for MERS-CoV
Human herpesvirus 6 (HHV-6)	Ganciclovir for immunocompetent patients Foscarnet or ganciclovir for immunocompromized hosts with severe infections	Supportive care	None
Human parainfluenza viruses (HPIVs)	None	Supportive care Corticosteroids for croup Conflicting results for ribavirin and IVIG	Infection control
Human polyomavirus (BK, JC virus)	None	Supportive, reduction of immunosuppression JC virus: ART in HIV patients	None
Measles	Ribavirin may be used in severe cases—not enough data for its efficacy	Supportive care, vitamin A	Vaccination, infection control, post-exposure prophylaxis in unvaccinated contacts (vaccine or human immunoglobulin)
Poliovirus/cVDPV		Early intensive physical therapy	Vaccine (IPV, OPV)
Rabies Lyssavirus	None	Supportive care	Vaccine
Respiratory syncytial virus (RSV A,B)	Ribavirin, palivizumab	Supportive care Glucocorticoids for immunocompromized patients	Infection control Palivizumab prophylaxis in infants and children when indicated
SARS-CoV-2	Remdesivir	Dexamethasone (strong recommendation for hospitalized patients requiring oxygen or under MV), antithrombotic treatment Under investigation or EUA by FDA: Baricitinib, anti-SARS-CoV-2 monoclonal antibodies, convalescent plasma, anakinra, cell therapies, convalescent plasma, IVIG and other interleukin inhibitors, kinase inhibitors, interferon	Infection control Vaccination Repurposed agents are tested for SARS-CoV-2 (e.g., BCG vaccine, Hydroxychloroquine/Chloroquine)
Seasonal influenza (Influenza virus A: A/H1N1pdm09, A/H3N2, Influenza virus B: B/Yamagata, B/Victoria)	NAIs (oseltamivir, zanamivir, peramivir) Inhibitor of influenza cap-dependent endonuclease (baloxavir)	IVIG, convalescent plasma (further studies needed)	Infection control, annual tetravalent vaccination Post-exposure prophylaxis with oseltamivir or zanamivir when indicated
Varicella-zoster virus (VZV)	Nucleoside analogs (acyclovir, valacyclovir, famciclovir)	Corticosteroids for VZV vasculopathy	Vaccination, infection control, post-exposure prophylaxis with vaccination, immunoglobulin or antiviral therapy
Variola virus (smallpox)^**^	Tecovirimat	Supportive care	Vaccination
**Supportive treatment and infection control only for the following viruses:**			
Human bocavirus (HBoV) Human metapneumovirus (HMPV) Human rhinoviruses (HRhVs 1–4) HCoVs HHV-7 Murray Valley encephalitis virus Powassa virus Saint Louis encephalitis virus Tick-borne encephalitis virus West Nile virus		Zika virus La Crosse / California encephalitis virus Toscana virus Rift Valley Fever virus Colorado tick fever Lassa virus Marburg virus Hantavirus (New World strains causing HCPS) Mimivirus (APMV) Nipah Virus	

* *No new cases reported since 2004*.

** *No new cases reported since 1977*.

*AFP, acute flaccid paralysis; APMV, acanthamoeba polyphaga mimivirus; ART, anti-retroviral therapy; BCG, Bacillus Calmette-Guerin; cVDPV, circulating vaccine-derived poliovirus; EV, enterovirus; EUA, emergency use authorization; HCPS, Hantavirus Cardiopulmonary Syndrome; IFNα, interferon-α; IPV, inactivated poliovirus vaccine; IV, intravenous; IVIG, intravenous immunoglobulin; MERS-CoV, middle east respiratory syndrome-related coronavirus; MV, mechanical ventilation; NAI, neuraminidase inhibitor; OPV, oral polio vaccine; SARS-CoV-2, severe acute respiratory syndrome-related coronavirus 2; SARS-CoV, severe acute respiratory syndrome-related coronavirus*.

The effects of CMV reactivation on adverse clinical outcomes in immunocompetent critically patients have been associated with higher blood viral loads (208). Although no studies have robustly responded to whether and when CMV reactivation should be treated in this patient population (209), it has been suggested that therapy should be administered if CMV reactivation is associated with organ-involvement and/or if high viral loads are detected (≥10^3^ copies/mL) (208).

Two randomized controlled trials assessing the use of prophylactic or preemptive treatment with aciclovir for HSV reactivation in mechanically ventilated patients, found that aciclovir administration did not improve clinical outcomes (207). As for HSV, two randomized controlled studies (the GRAIL-trial and the CCCC-trial) evaluating the efficacy of preemptive treatment for CMV reactivation in seropositive patients, failed to show benefit in IL-6 levels changes and in clinical outcomes, respectively (209–211). The GRAIL-trial, however, demonstrated that ganciclovir group had significantly more ventilator-free days in the sepsis subgroup analysis (209, 211). Based on expert-opinion, it has been suggested that preemptive treatment may be given in patients either at high-risk for HSV or CMV reactivation or in those who already have reactivation but at an early stage, before the advent of clinically evident infection (208). On the other hand, both aciclovir and ganciclovir have potentially serious adverse effects, thus the need for their administration in critically ill patients should be weighed against patient safety and cost-effectiveness (212).

### Specific Treatments and Vaccines

#### Antivirals

The cornerstone of severe influenza management is the prompt initiation of antivirals (for at least 5 days, but a 10-days course is generally recommended in severe pneumonia) combined with high level of supportive care [Table 7; (213)]. The recommended antivirals are neuraminidase inhibitors (NAIs), i.e., oseltamivir, zanamivir, and peramivir. Baloxavir, a novel inhibitor of the cap-dependent endonuclease activity, is currently recommended only for uncomplicated influenza; adamantanes are no longer recommended due to development of resistance (214, 215). Although high-dose oseltamivir (300 mg/day) had been proposed for critically ill adults with severe pneumonia, no studies have proven its superiority against standard dose (150 mg/day) (216, 217). NAIs reduce mortality in severe influenza patients irrespective of the time of initiation, but early initiation (i.e., within 48 h of symptoms onset) reduces significantly the mortality (218). Oseltamivir and zanamivir may also be effective against selected avian influenza strains, but more studies are needed.

There are still no effective antivirals available for the MERS-CoV infection; however, two combinations [lopinavir/ritonavir/interferon beta (IFNβ) and remdesivir/IFNβ] are currently being tested *in vivo* and in animal models (219). Data from randomized controlled trials examining remdesivir in COVID-19 patients, show that remdesivir might be effective in reducing the time to recovery in hospitalized adults, and based on these trials it is currently the only antiviral agent that has gained authorization by the U.S. Food and Drug Administration (FDA) for use in hospitalized patients requiring supplemental oxygen (220–224). However, in November 2020, the WHO issued a conditional recommendation against the use of remdesivir in hospitalized patients irrespective of disease severity, due to the lack of sufficient data supporting that remdesivir actually improves clinical outcomes, including mortality (225). As evidence from current trials is still limited, robust data for safe and efficacious antivirals against SARS-CoV-2 infection are urgently required (226).

Targeted antiviral agents for other respiratory viruses are limited (Table 7). Ribavirin (inhaled or intravenous) and intravenous immunoglobulin have been used with conflicting results in cases of HPIV-3 pneumonia in severely immunocompromised patients (227). Although supportive care is the mainstay of treatment of RSV infection, inhaled (or oral) ribavirin can be used in patients with significant risk factors for severe disease, like transplantation (228). For HMPV infection, treatment is mainly supportive, although ribavirin, intravenous immunoglobulin, monoclonal antibodies, and fusion inhibitory peptides against specific viral proteins have been used in isolated cases or in experimental models (229–234). Cidofovir and its oral prodrug brincidofovir are administered in severe HAdV infections (164, 235–237).

In contrast to respiratory infections, targeted antivirals are available for major CNS pathogens like HSV and VZV viruses (Table 7). Indeed, the mainstay of treatment for HSV encephalitis is aciclovir (10 mg/kg intravenously every 8 h), though dose adjustments are required for impaired renal function. The duration of treatment ranges from 14 to 21 days, and should be administered as early as possible. Similarly, treatment of choice for severe VZV CNS infection is intravenous aciclovir for 14–21 days, but in higher doses than those administered in HSV encephalitis [i.e., up to 15 mg/kg three times a day; (238)]. Of note, aciclovir can cause crystal nephropathy and acute renal failure in 12–49% of patients, with those in ICU being at greater risk (239). Treatment of CMV encephalitis consists of dual therapy with foscarnet and ganciclovir, as no survival benefit has been observed with either agent alone; duration of treatment is 3 weeks for immunocompetent patients and 6 weeks for those with immunodeficiency (240). Secondary prophylaxis is required until immune restoration (CD4+ >100 cells/ml) in AIDS patients and antiretroviral treatment should start as soon as possible (241).

Finally, patients with severe acute HBV hepatitis should be treated with a nucleos(t)ide analog reverse-transcriptase inhibitor like entecavir or tenofovir, while ribavirin may be considered for severe acute HEV hepatitis (242, 243).

#### Corticosteroids

Corticosteroids have been used in various severe viral infections as immunomodulators abrogating the cytokine-release syndrome effects, with conflicting outcomes and results. In severe influenza, corticosteroids are associated with unfavorable outcomes like increased mortality and prolonged viral shedding as well as increased incidence of bacterial superinfections and possibly prolonged duration of mechanical ventilation (78, 244); thus, their administration is not recommended in severe flu (213).

In contrast to influenza trials that failed to show benefit in mortality and improved outcomes, the Randomized Evaluation of COVid-19 thERapY (RECOVERY) trial for COVID-19 treatment reported that low dose dexamethasone (6 mg once per day either orally or by intravenous injection for 10 days) along with standard of care reduced significantly the incidence of death among mechanically ventilated patients (29.3 vs. 41.4%; rate ratio, 0.64; 95% CI, 0.51–0.81) and among those on supplemental oxygen (23.3 vs. 26.2%; rate ratio, 0.82; 95% CI, 0.72–0.94) compared to those who received standard treatment, thus being the first agent that was found to reduce mortality in moderate, severe and critical COVID-19 cases (245). However, no mortality benefit was found in patients not requiring oxygen support (245). Additionally, the CoDEX trial found that among patients with moderate and severe COVID-19-associated ARDS, the intravenous administration of dexamethasone (in higher doses than RECOVERY trial) resulted in a statistically significant increase of the number of ventilator-free days over 28 days (246). Nonetheless, randomized controlled trials using other corticosteroids, such as methylprednisolone and hydrocortisone, failed to show similar survival benefits (247, 248). As of January 2021, dexamethasone is the only therapeutic agent being strongly recommended for COVID-19 patients requiring (non-invasive) supplemental oxygen or mechanical ventilation by the international stakeholders (220, 249). In other viral infections, corticosteroids are used only for certain indications, like in HPIV-induced croup to reduce airway edema or in VZV-induced CNS vasculopathy, whereby a short course of high-dose corticosteroids is recommended (46). The use of corticosteroids in HSV encephalitis is debatable, but it may be useful in selected cases with vasogenic edema and mass effect (46). Intravenous immunoglobulin, and in refractory cases methylprednisolone, have been used in refractory cases of enteroviral encephalitis (54, 250).

#### Blood-Derived Products and Cell Therapies

Passive immunization with antibodies purified from pooled sera, convalescent plasma and hyperimmune intravenous immunoglobulin has been used as a last resort measure in severe viral infections, when other treatment options have failed. Hyperimmune intravenous immunoglobulin from convalescent plasma was associated with reduced mortality in severe A/H1N1pdm09 infection in a randomized controlled trial, but its use is not universally recommended yet (251).

Results are pending for the safety and efficacy of convalescent plasma, SARS-CoV-2 immunoglobulin, non-SASRS-CoV-2 intravenous immunoglobulin, and mesenchymal stem cell therapies in COVID-19 infection; due to the lack of sufficient evidence, these agents are either non-recommended outside the context of clinical trials or have yet to receive no specific recommendations for or against their use (226, 252).

Finally, while treatment is mainly supportive in the WNV infection, intravenous immunoglobulin—either conventional or from donors with anti-WNV antibodies—has been used successfully in some cases, but there is no universal recommendation as yet (160).

#### Immunotherapies

As pathogenesis of severe and critical COVID-19 disease has been linked primarily to cytokine storm syndrome, many research efforts are made with trials testing various immunomodulatory agents like tocilizumab, sarilumab, anakinra, Janus kinase (JAK) inhibitors, adalimumab, anti-programmed cell death protein 1 monoclonal antibodies, bevacizumab, ixekizumab, eculizumab, human recombinant IL-2, inhibitors of NLRP3 inflammasome activation, Janus kinase inhibitors, fingolimod, a recombinant fusion protein targeting an immune pathway checkpoint (CD24Fc), leflunomide, thalidomide, and colchicine (226). Baricitinib, a JAK 1 and 2 selective inhibitor, is approved by the FDA for emergency use in combination with remdesivir in hospitalized COVID-19 patients aged ≥2 years old who require supplemental oxygen, invasive mechanical ventilation or extracorporeal membrane oxygenation (253). Additionally, three anti-SARS-CoV-2 monoclonal antibodies (bamlanivimab and the combination of casirivimab plus imdevimab) have received authorization by the FDA for emergency use in outpatients with COVID-19 who are at risk for disease progression (220).

Palivizumab (15 mg/kg intramuscularly per month), a monoclonal anti-RSV antibody, has substituted immunoglobulin products with high anti-RSV antibody titers (RSV-IGIV) in the prevention of RSV infection in high-risk infants (254, 255). Additionally, various monoclonal antibodies against other viruses like CMV, Influenza, HIV, Ebola and Rabies, are currently under development (256).

#### Vaccinations

Vaccines are the most important preventative measures for many serious viral infections (Table 7). Annual vaccination is of utmost importance for influenza prevention and is recommended for individuals over the age of 6 months without contraindications (257). Although several vaccines are under development for MERS-CoV, none has been licensed yet (258). On the contrary, immense research has led to the development of more than 240 vaccine candidates against SARS-CoV-2; as of January 2021, at least 8 effective SARS-CoV-2 vaccines have been officially approved in numerous countries worldwide (259). Besides the unparalleled speed of vaccine development (<1 year after the onset of COVID-19 pandemic), it is also quite impressive that this pandemic incited the utilization of next generation vaccine platforms, such as the messenger RNA and non-replicating viral vector-based vaccines, that have already been launched for public use (259, 260).

Live oral vaccines are highly effective in reducing the risk of respiratory HAdV infection, but are available only within the U.S. military settings (261). Zoster vaccination in adults has been associated with reduced incidence of herpes zoster, but the data for its efficacy in preventing CNS reactivation and complications are limited (262). Finally, post-exposure prophylaxis with rabies vaccine and immunoglobulin can prevent rabies in almost 100% of the exposed individuals (263, 264).

## Conclusion

Viral infections are important determinants of adverse outcomes especially among critically ill patients. Their clinical significance as pathogens, although largely disregarded in the past, is now increasingly recognized especially due to the unprecedented COVID-19 pandemic. The advent of more sensitive and rapid molecular tools has changed the landscape in microbiological diagnosis and in identification of fastidious pathogens, including viruses that are increasingly isolated among critically ill patients. The interplay between direct viral organ evasion and the effects of the hosts' immune system dysregulation defines the clinical severity and the outcomes of viral infections. As effective therapies are currently lacking for the majority of viruses causing severe infections, more clinical trials are urgently needed to determine whether novel antivirals, steroids, or immunomodulators can impact better clinical outcomes in viral infections in the ICU.

## Author Contributions

PF, EK, and CM contributed in the collection of the data, wrote parts the first draft, and the subsequent revisions of the manuscript. TK and ST critically revised and corrected the manuscript. All authors read and approved the submitted manuscript version.

## Conflict of Interest

The authors declare that the research was conducted in the absence of any commercial or financial relationships that could be construed as a potential conflict of interest.
